# DosR’s multifaceted role on *Mycobacterium bovis* BCG revealed through multi-omics

**DOI:** 10.3389/fcimb.2023.1292864

**Published:** 2023-11-21

**Authors:** Yingying Cui, Guanghui Dang, Hui Wang, Yiyi Tang, Mingyue Lv, Siguo Liu, Ningning Song

**Affiliations:** ^1^ State Key Laboratory for Animal Disease Control and Prevention, Division of Bacterial Diseases, Harbin Veterinary Research Institute, Chinese Academy of Agricultural Sciences, Harbin, China; ^2^ School of Life Science and Technology, Weifang Medical University, Weifang, China; ^3^ Weifang Key Laboratory of Respiratory Tract Pathogens and Drug Therapy, Weifang, China

**Keywords:** *Mycobacterium bovis* BCG, DosR, transcriptomics, proteomics, metabonomics

## Abstract

*Mycobacterium tuberculosis* (Mtb) is an intracellular bacterium that causes a highly contagious and potentially lethal tuberculosis (TB) in humans. It can maintain a dormant TB infection within the host. DosR (dormancy survival regulator) (Rv3133c) has been recognized as one of the key transcriptional proteins regulating bacterial dormancy and participating in various metabolic processes. In this study, we extensively investigate the still not well-comprehended role and mechanism of DosR in *Mycobacterium bovis* (*M. bovis*) Bacillus Calmette-Guérin (BCG) through a combined omics analysis. Our study finds that deleting DosR significantly affects the transcriptional levels of 104 genes and 179 proteins. Targeted metabolomics data for amino acids indicate that DosR knockout significantly upregulates L-Aspartic acid and serine synthesis, while downregulating seven other amino acids, including L-histidine and lysine. This suggests that DosR regulates amino acid synthesis and metabolism. Taken together, these findings provide molecular and metabolic bases for DosR effects, suggesting that DosR may be a novel regulatory target.

## Introduction

Tuberculosis (TB) remains a global health concern, affecting an estimated one-third of the world’s population and causing 9 million deaths annually (Harding, 2020). The causative agent, *Mycobacterium tuberculosis* (Mtb), is an ancient intracellular pathogen capable of evading the immune system and persisting within macrophages for extended periods of time (Chakaya et al., 2021). To survive, Mtb must adapt to the antimycobacterial granuloma microenvironment, which is characterized by hypoxia and nutrient scarcity. Gaining a more in-depth understanding of this adaptation process requires a gene expression data analysis at multiple regulatory levels.

In response to Mtb infection, macrophages and T cells limit the growth of bacteria by aggregating at the site of invasion and creating granulomas, which release interleukin 12 (IL-12), tumor necrosis factor (TNF), interferon-γ (IFN-γ), and reactive nitrogen intermediates (Vesosky et al., 2010). In this microenvironment, Mtb has limited oxygen and nutrient availability while being exposed to a potent antimicrobial nitric oxide (NO) and low pH (Shiloh et al., 2008). Oxygen tension impacts the growth, transcription, and metabolism of Mtb. Hypoxia induces a variety of specific genes vital for Mtb infection and persistence, such as *dosR* (dormancy survival regulator) (Rustad et al., 2009). The *dosR* gene is activated in microenvironments with low oxygen and elevated Nitric oxide (NO), Carbonic oxide (CO), and reactive oxygen species (ROS) levels. Such conditions have been supported as conducive and necessary for Mtb survival *in vitro* by several studies on guinea pigs and rabbits (Park et al., 2003). The DosRST system is a two-component control system that includes two sensor kinases, DosS, and DosT. These kinases detect signal molecules and activate their kinase activities, thereby relaying signals to DosR (Roberts et al., 2004; Honaker et al., 2009; Kim et al., 2010). In response to the environmental stress, Mtb develops a robust adaptation program by inducing the DosR regulon containing 48 genes. Recent studies have indicated that the DosR protein can participate in the regulation of arginine and the immune response (Voskuil et al., 2004; Gao et al., 2019; Cui et al., 2022). Furthermore, amino acid biosynthesis, which utilizes intermediates of the tricarboxylic acid cycle, is essential for Mtb’s survival (Hasenoehrl et al., 2019). Specific amino acids have been found to be correlated with Mtb, as demonstrated by *in-vivo* and *in-vitro* studies. For example, L-Arginine serves as a precursor for the synthesis of NO, while the arginine metabolism significantly influences the macrophage-mediated killing of Mtb (Qualls et al., 2010; Tiwari et al., 2018). Furthermore, the modulation of tryptophan metabolism has been suggested as a novel therapeutic approach for adjuvant TB treatment (Crowther and Qualls, 2020). Additionally, *de-novo* synthesis of histidine serves to protect mycobacteria from IFN-γ–mediated histidine starvation (Dwivedy et al., 2021).

The changes in gene expression induced by the DosR regulator and its downstream effects have been comprehensively analyzed (Gautam et al., 2015). Previous studies focused primarily on the effects of DosR under hypoxic conditions, leaving the impact of deleting DosR on amino acid metabolism under aerobic conditions largely unexplored. In this study, we specifically examined the differences in gene expression and protein abundance between the wild-type (WT) BCG and BCGΔDosR strains. Furthermore, we performed targeted metabolomic analyses to investigate potential roles of amino acids in Mtb’s adaptive survival in the infected host.

## Methods

### Bacterial strains and culture conditions

*Mycobacterium bovis* BCG Tokyo 172 and BCGΔDosR strains were cultured in Middlebrook 7H9 broth medium (BD Biosciences, San Jose, CA, USA) at 37°C. The medium was supplemented with 0.05% Tween 80 (v/v) (Amresco, Solon, OH, USA), 0.2% glycerol (v/v) (Sigma-Aldrich, Shanghai, China), and 10% OADC (v/v) (BD). When the OD_600nm_ reached 0.6, bacterial cells were collected, washed with phosphate buffered saline, and used for further analysis. Three or six replicates of each experiment were prepared for data collection.

### Purification of recombinant DosR protein

The expression and purification of DosR recombinant protein were performed as previously described (Cui et al., 2022). Specific primers (**Supplementary Material S1**, **Supplementary Table S1**) were used to amplify the *dosR* gene and clone it into the pET-22b vector to construct the recombinant pET-22b-*dosR* plasmid, using the genome of the H37Rv strain as a template. The DosR protein was generated by growing the transformed *Escherichia coli* BL21 (DE3) cells in 200 mL of LB broth and inducing the expression with 1 mM isopropyl β-D-1-thiogalactopyranoside supplementation. The cells were harvested and then sonicated in a lysis buffer (20 mM tris-HCl, 150 mM NaCl, and 10% glycerol, pH 8.0). The resulting supernatant was purified using affinity chromatography to isolate the DosR protein.

### Construction of DosR-deletion mutant in the BCG strain

The knockout of *dosR* was achieved by homologous recombination, following the procedure described in a previous study (Bardarov et al., 2002). Polymerase chain reaction (PCR) amplified the left and right homologous arms of *dosR* from BCG’s genome. The allelic exchange substrate (AES) was constructed by linking four fragments, including the *hygromycin* and the *sacB* gene, following the digestion of the amplified DNA with *Van91I*. The cassette in the suicide plasmid phAE159 was replaced with the AES containing the *PacI* site, followed by *in-vitro* packaging. The hygromycin resistance gene was used as a selection marker to facilitate the targeted deletion of DosR in the BCG strain (BCGΔDosR). We then electroporated the packaged *dosR-phAE159* plasmid into *Mycobacterium smegmatis* to produce high-titer phage lysates. These lysates were used to infect BCG strains, resulting in the separation of *dosR* deletion strains. The required primers used and identification of BCGΔDosR in the assay were shown in **Supplementary Material S1** and **Supplementary Table S1**.

### Transcriptomic analysis

RNA-seq was performed on six samples, including three biological replicates, each for WT BCG and BCGΔDosR. Cultures were grown in 7H9 medium at 37°C with shaking (100 rpm) until reaching OD_600_ = 0.8. Total RNA was extracted using TRIzol Reagent (Amresco, Framingham, US) and processed with Truseq™ stranded RNA sample prep kit (Qiagen, Hilden, Germany). Ribosomal RNA was removed using a Ribo-Zero Magnetic kit. mRNA was fragmented following the TruSeq™ stranded RNA sample prep kit instructions after cDNA synthesis. During the second strand cDNA synthesis, deoxyuridine triphosphate replaced deoxythymidine triphosphate, followed by uracil-DNA glycosylase digestion. The resulting cDNA library underwent PCR enrichment for 15 cycles and purification with 2% agarose gel. Library quantification was performed using TBS380 (Picogreen) and sequenced on the HIseq platform.

Sequencing raw data was processed using Base Calling and Trimmomatic software for quality assessment and base distribution analysis. UMI redundancy was removed, and reads matching ribosomal RNA (rRNA) were excluded after comparison with the Rfam database. Subsequently, the sequencing data for each sample were mapped to the reference genome using Rockhopper, specialized software for prokaryotic transcriptome analysis. Gene expression levels were measured with Rockhopper. Local regression identified gene expression patterns, and statistical comparison utilized the negative binomial distribution model, providing *p*-values. Finally, the Benjamini–Hochberg multiple testing correction was applied to these *p*-values to obtain the *Q*-values for significant gene comparisons.

### Proteomic analysis

Protein expression in three biological replicates of WT and mutant *ΔdosR* samples was quantified. The proteins from the WT BCG and BCGΔDosR were prepared by sonication in five volumes (v/w) of an isolation buffer containing 4% SDS, 0.1 M Tris-HCl, 10 mM DTT, 8 M urea, and 1% protease inhibitor cocktail (pH = 8.0). All samples were centrifuged at 20,000 ×g for 10 min at 4°C. The supernatant was collected and its concentration was determined using bicinchoninic acid protein assay kit (Thermo Scientific) following the manufacturer’s instructions. Subsequently, approximately 200 g of protein was reduced with DTT at 37°C for 2h, and 20 mM iodoacetamide (IAM, Sinopharm) was used to block sulfhydryl groups in the dark for 1h. The prepared protein sample was diluted (V/V, 1:5, Sinopharm) with 50 mM NH_4_HCO_3_ and digested overnight at 37°C with trypsin (Sigma-Aldrich). The reaction was stopped with formic acid (Sigma-Aldrich), and the products were desalted and vacuum-dried for Tandem Mass Tag (TMT) labeling and analysis.

The mass spectrum data obtained were processed using Proteome Discoverer software, and the mass spectrum was analyzed (Saleh et al., 2019). The related parameters were: precursor ion mass range: 350-8000 Da, and a signal-to-noise ratio S/N threshold of 1.5. The peptide data obtained from mass spectrometry analysis were further investigated using Proteome Discoverer software (PD) (version 1.4.0.288, Thermo Fisher Scientific). Subsequently, the spectra extracted using PD were searched by Mascot (version 2.3.2, Matrix Science). A quantitative analysis was then performed, based on the Mascot search results and the spectra screened in the initial evaluation stage. The resulting map was searched in the UniProt database using Mascot. Subsequently, it was subjected to both qualitative and quantitative analyses. For the quantitative assessment, the following parameters were applied: Protein Ratio Type: Median, Protein Quantification: Use Only Unique Peptides, Normalization Method: None, *p*-value: <0.01, and a threshold of Multiples of the Difference: > 1.2-fold change.

### UHPLC-MS/MS analysis of amino acids

The amino acid concentrations in WT BCG and BCGΔDosR were determined using high resolution ultra-performance liquid chromatography–mass spectrometry (UHPLC-MS/MS) as previously described (Song et al., 2020). Both WT BCG and BCGΔDosR strains were cultured until they reached the logarithmic growth phase. Subsequently, the cell pellets (approximately 100 mg) were ground in liquid nitrogen and extracted with 600 μL of extraction solvent (acetonitrile-methanol-water, 2:2:1). An 80-μL aliquot of the resulting supernatant was transferred and used for UHPLC-MS/MS analysis.

The UHPLC separation was carried out at 35°C, using an Agilent 1290 Infinity II series UHPLC System (Agilent Technologies, Santa Clara, CA, USA) with the Amide column (100 mm × 2.1 mm, 1.7 μm) attached. Analytes were separated by chromatography, using the gradient elution program with two solvents system: solvent A (water containing 0.1% formic acid) and solvent B (acetonitrile containing 0.1% formic acid) at a flow rate of 0.3 mL/min. Mass spectral analysis was performed on an Agilent 6460 triple quadrupole mass spectrometer. Typical ion source parameters were as follows: capillary voltage = +4000/−3500 V, nozzle voltage = +500/−500 V, gas (N_2_) temperature = 300°C, gas (N_2_) flow = 5 L/min, sheath gas (N_2_) temperature = 250°C, sheath gas flow = 11 L/min, nebulizer = 45 psi.

Agilent MassHunter Workstation Software (B.08.00, Agilent Technologies, PaloAlto, CA, US) was employed for Multi reaction monitoring mode (MRM) data acquisition and processing. The precision of the quantification was measured as the relative standard deviation, determined by injecting analytical replicates of a quality control (QC) sample. The accuracy of quantification was determined by calculating the analytical recovery of the QC sample, expressed as a percentage: [(mean observed concentration)/(spiked concentration)] × 100%.

### ROS and TUNEL assays

For ROS and TdT-mediated dUTP Nick-End Labeling (TUNEL) assays, WT BCG and BCGΔDosR strains were grown until the culture reached an OD_600_ of 0.6–0.8, followed by three washes in phosphate-buffered saline solution (PBST). After centrifugation, the pellet was resuspended with 1 mL of PBST, and the aliquot was analyzed by incubation with 1 μM of ethidium dihydrogen (Sinopharm) for 30 min, protected from light. All prepared samples were confirmed by flow cytometry. For the TUNEL analysis, all operations were performed with an *In Situ* Cell Death Kit, following the manufacturer’s instructions (Roche, Basel, Switzerland).

### Electrophoretic mobility shift assay analysis

Electrophoretic mobility shift assay (EMSA) was performed to verify the binding ability of DosR with target promoters. The Cy-5 labeled DNA substrates were amplified by PCR, using specific primers (**Supplementary Material S1**, **Supplementary Table S1**) from *M. tuberculosis* H37Rv genome. For the EMSA test, DosR and labeled fragments were incubated in binding buffer (20 mM tris-HCl, 150 mM NaCl, 1 mM DTT, and 5% glycerol) at 25°C for 30 min. After incubation, the complex was analyzed on a 6% nondenatured polyacrylamide gel in 0.5 × Tris-Borate-EDTA buffer at 150 v for 3h. Subsequently, the gel was analyzed and imaged using a typhoon scanner (GE Healthcare).

## Results

### Mapping information

To identify the potential targets regulated by DosR, we conducted RNA-seq and proteomic analyses to compare the gene expression in BCG and BCGΔDosR strains. We quantified 42,132,713 RNA fragments and 45,775 peptides, resulting in the identification of 4,088 transcripts and 3,176 proteins. Notably, the most significant differences in the molecules’abundance were observed between the BCG and BCGΔDosR strains, involving 104 transcripts and 179 proteins (**Supplementary Tables S2, S3**). In BCGΔDosR strain, five genes were upregulated, while 99 were downregulated, compared to BCG.

To generate a high confidence list of genes whose expression levels differ between the BCG and BCGΔDosR strains, we compared the RNA-seq and proteomic datasets. Among the proteins and transcripts measured in both experiments, we identified a large overlap of eight genes. These genes were consistently and significantly downregulated in BCGΔDosR (**Table 1**), as evidenced by both proteomics and RNA-seq data. Results obtained from qPCR for eight genes were consistent with the transcriptomic data (**Supplementary Material S1**, **Supplementary Figure S2**). This finding underscores the intricate adaptability of DosR in response to environmental changes and its vital role in supporting metabolic processes.

**Table 1:** Common hits to RNA-seq and proteomics.

Gene	Description	*P*-value protein	Log-fold change protein	*P*-value RNA	Log-fold change RNA
JTY_RS00440 (Rv0079)	Putative regulatory protein	0.04933222	−0.988339986	< 0.001	−5.58764
JTY_RS00445 (Rv0080)	Pyridoxamine 5’-phosphate oxidase	0.03602813	−0.587570464	< 0.001	−5.9259
JTY_RS03920 (Rv0744c)	Transcriptional regulatory protein	0.03755898	−0.609750866	2.69E-39	−1.11916
JTY_RS10505 (Rv2028c)	Universal stress protein	0.04208272	−0.85164874	< 0.001	−4.64263
JTY_RS16225 (devR)	Response regulator DosR	0.01438424	−2.495145364	< 0.001	−8.997713919
JTY_RS16220 (devS)	Oxygen sensor Histidine kinase DevS	9.35E-05	−1.2236737	1.93E-86	−1.991939625
JTY_RS13655 (Hrp1)	Hypoxic response preotein 1	0.02973263	−0.697167208	< 0.001	−2.771884
JTY_RS10520 (hspX/acr)	Alpha-crystallin	0.03599509	−1.290482831	< 0.001	−5.01959089

Combined transcriptomic and proteomic analysis confirmed that eight genes were significantly downregulated in BCGΔDosR. JTY_RS00440 and Rv0079 are highly homologous, JTY_RS00445 represents Rv0080, JTY_RS03920 represents Rv0744c, and JTY_RS10505 represents Rv2028c.

### Transcriptomic analysis of WT and mutant *ΔdosR*

**Figure 1:**
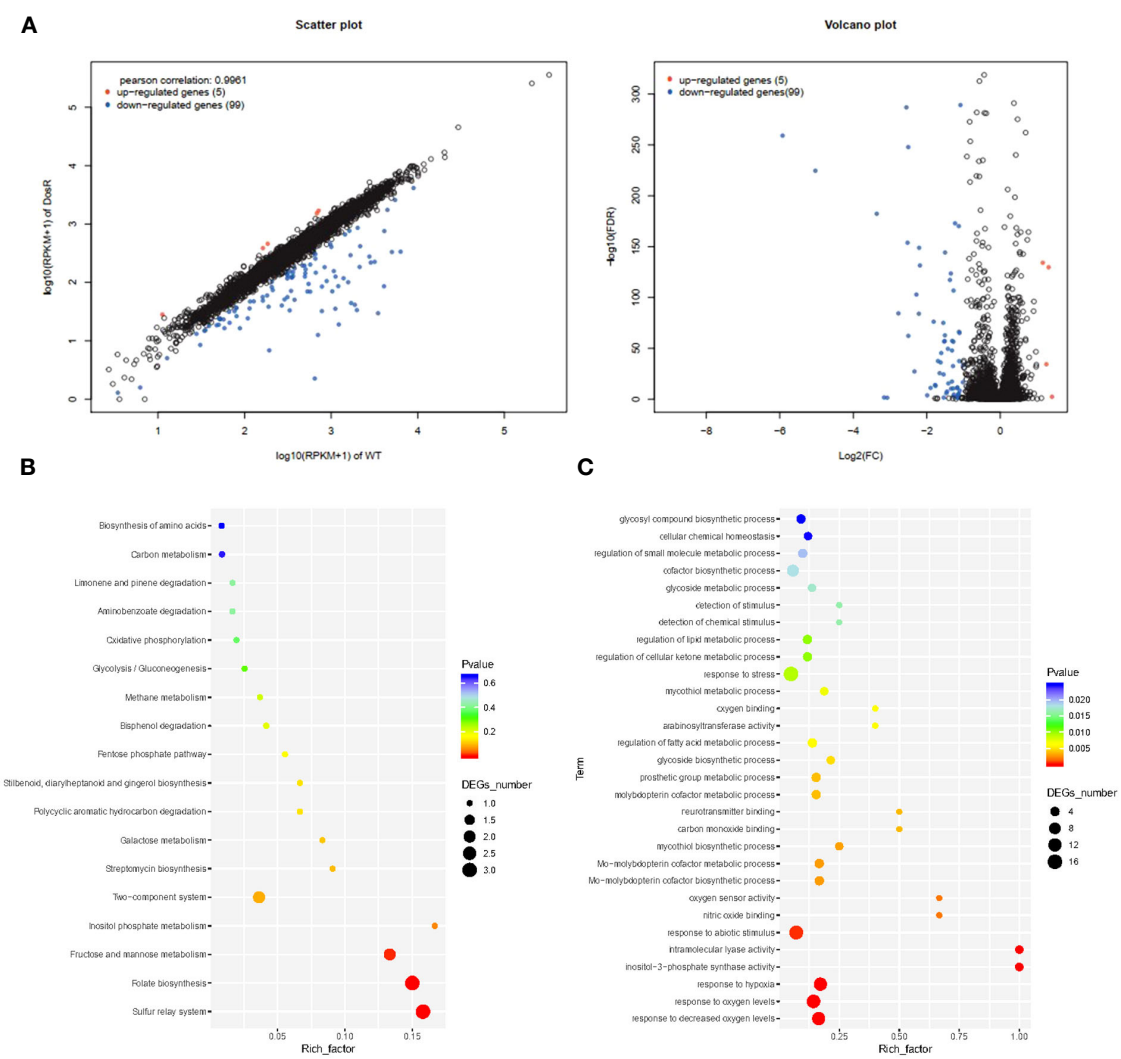
Transcriptome analysis. **(A)** Volcano plot showing the relative abundances of transcripts (mutant BCGΔDosR vs. WT BCG). **(B)** KEGG enrichment analysis. The horizontal axis represents the enrichment factor. The ordinate represents the function enriched by the GO term, and the size of the circle represents the enriched genes. The spectrum from blue to red represents uncorrected *P*-values. **(C)** GO enrichment analysis. Plots of GO term enrichments (*P* < 0.025). The horizontal axis represents the enrichment factor (the ratio of the number of differential genes enriched to a certain GO term to the number of background genes obtained by sequencing); the circles, the number of differential genes for this function. The color spectrum from blue to red represents the *p*-value.

To analyze gene expression differences between the WT and mutant *ΔdosR*, we performed Illumina sequencing on cDNA libraries derived from three biological replicates. More than 12 million reads were detected in each sample, with clean reads constituting more than 88%. In each replicate, we identified over 11,680,262 perfectly matched reads. A total of 104 genes were differentially expressed between WT and mutant *ΔdosR*. In comparison to WT, five genes were upregulated, while 99 genes were downregulated in the mutant *ΔdosR*, as shown in **Figure 1A**.

To assess the potential functions of differentially expressed genes, we assigned gene ontology (GO) categories. Subsequently, we performed significant transcriptional enrichment analysis between BCG and BCGΔDosR strains using the Goatools program (https://github.com/tanghaibao/GOatools). A total of 64 GO terms were identified as enrichments, encompassing 39 biological processes, 13 cellular components, and 22 molecular functions (**Figure 1B** and **Supplementary Table S4**). The most enriched terms were associated with oxygen, including the responses to varying oxygen levels (GO: 0070482), decreased oxygen levels (GO: 0036293), and hypoxia (GO: 0001666). The differentially expressed genes between BCG and BCGΔDosR were found to participate in 18 KEGG pathways (**Figure 1C**). Among the three pathways, the sulfur relay system, folate biosynthesis, and the two-component system were significantly enriched, as indicated by the results of the KOBAS analysis (http://kobas.cbi.pku.edu.cn/home.do) (**Supplementary Table S5**). Rv2029c (JTY_RS10510) and Rv0327c (JTY_RS01725) have been identified as participants in several physiological processes, including carbon metabolism and amino acid biosynthesis. Additionally, several transcriptional regulators, such as Rv2250c (JTY_RS11670), Rv1994c (JTY_RS10315), and Rv0047c (JTY_RS00260) were significantly downregulated in BCGΔDosR.

### Proteomic analysis of WT and mutant *ΔdosR*

**Figure 2:**
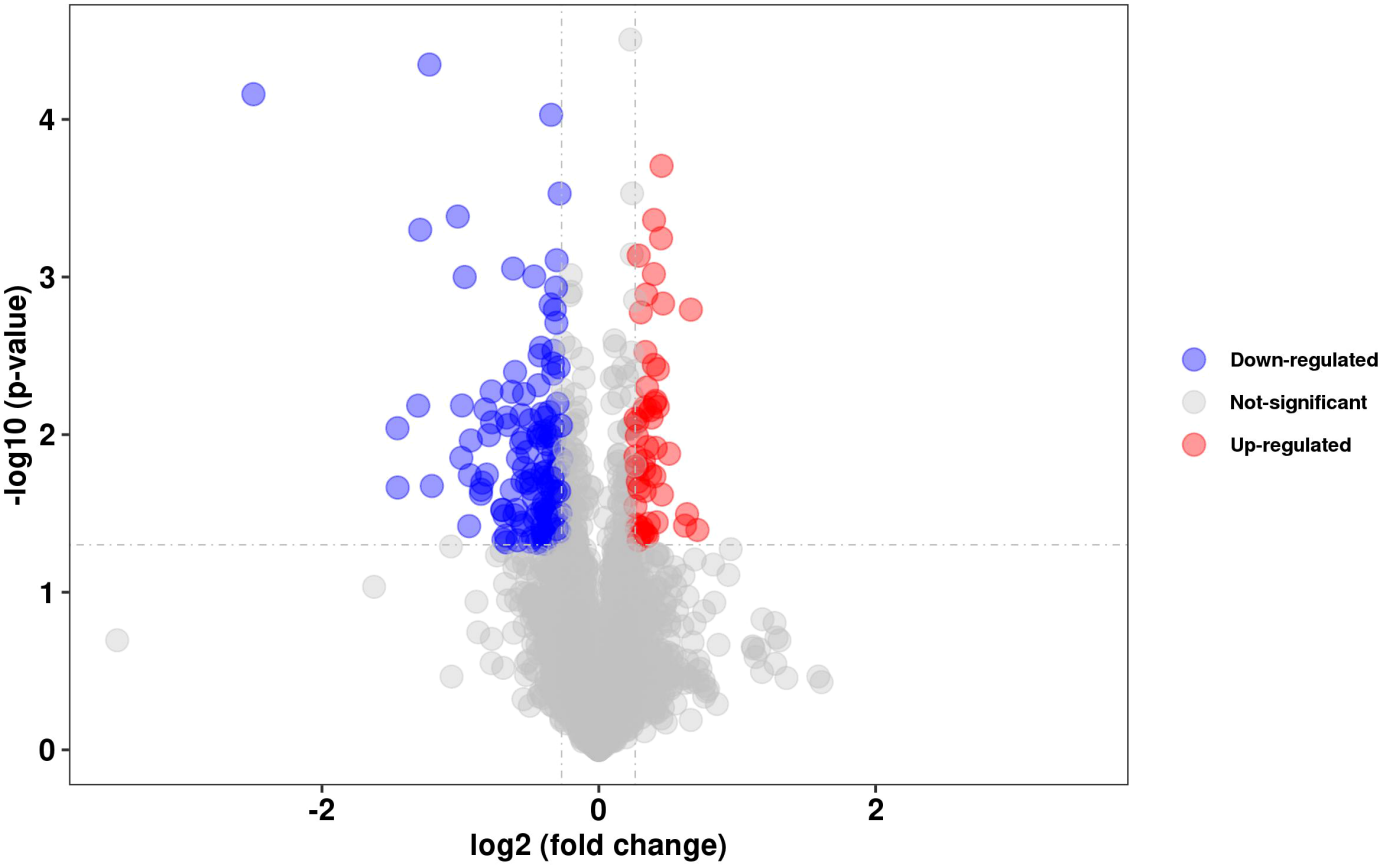
Proteomics analysis. Volcano plots showing the relative abundances of proteins (BCGΔDosR vs. WT BCG), *P*-VALUE < 0.05 and FOLD CHANGE < 0.83 or FOLD CHANGE > 1.2., FDR ≤ 0.05 & |log_2_(FC)| ≥1.

To evaluate the effect of DosR on the expression of other proteins, we compared the proteomic data of BCG and BCGΔDosR. We identified 179 proteins with significantly different gene expression levels between the two strains. One hundred twenty-six proteins were downregulated, while 53 proteins were upregulated, compared to the WT BCG (**Figure 2**). Notably, the DosR protein was significantly downregulated in the mutant BCGΔDosR. According to GO annotations, the differentially identified proteins were classified functionally into 33 categories (**Supplementary Table S6**). Additionally, based on the KEGG pathway analysis, we identified two enriched pathways. These pathways are the “two-component system” and “amino sugar and nucleotide sugar metabolism”. The TrcR-Trcs two-component regulatory system was the most significantly enriched pathway involved in amino sugar and nucleotide sugar metabolism. Additionally, the *rv2558* gene, previously reported to be upregulated in carbon-starvation conditions, exhibited higher gene expression in the deletion strain. To further characterize potential interactions between DosR and other proteins, we analyzed the binding network using the STRING database (**Supplementary Table S7**). In addition, we summarized the homologous genes between BCG and Mtb H37Rv strain in order to facilitate the homologous gene search (**Supplementary Table S8**). The results of the proteomic analysis indicate that DosR may be involved in more complex regulatory activities by modulating downstream transcription factors and triggering a cascade of reactions.

### Metabolism of amino acids

**Figure 3:**
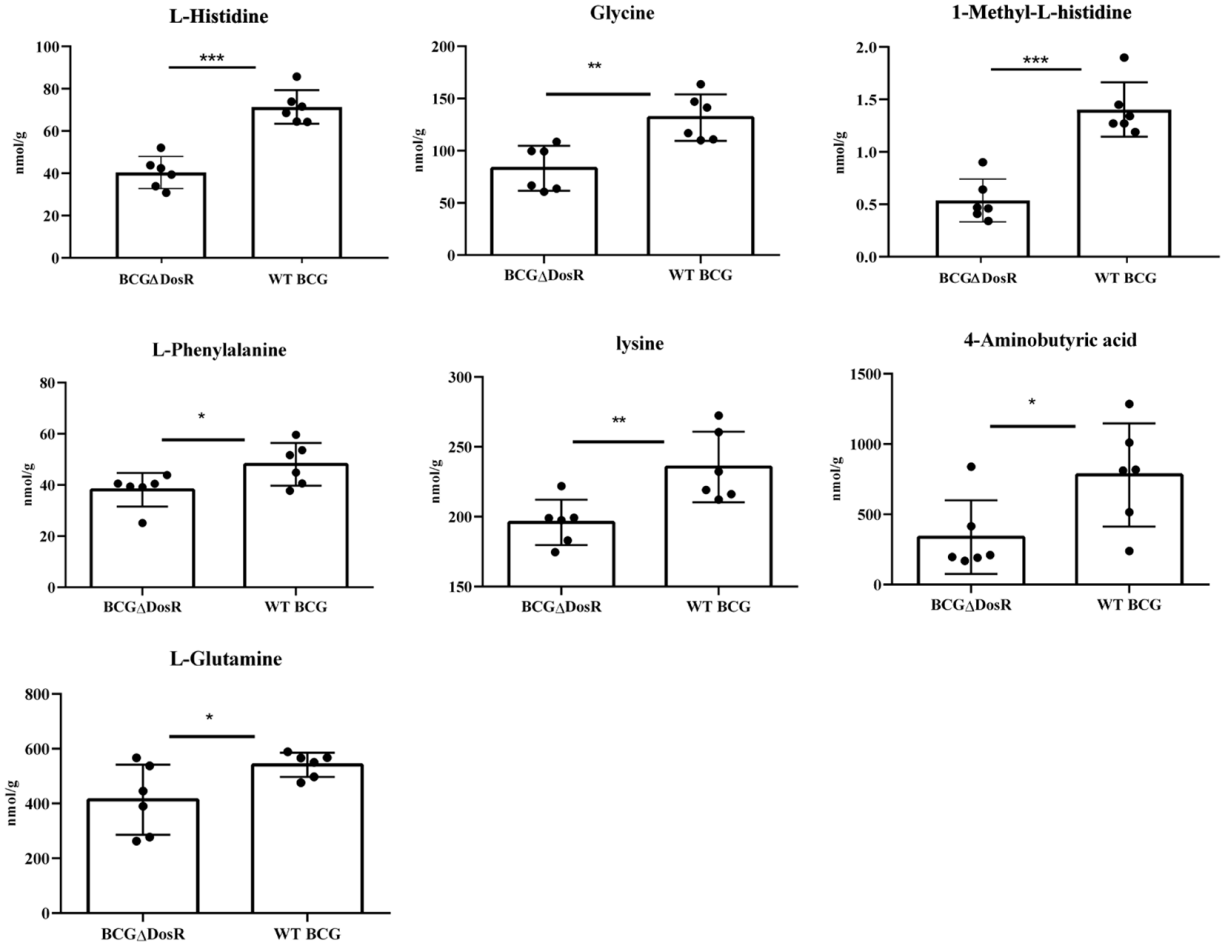
Amino acid metabonomic analysis. The concentration of amino acids in wild type BCG (WTBCG) and DosR deletion strains (BCGΔDosR) was analyzed by targeted metabonomics. The concentration of histidine, lysine, and seven other kinds of amino acids decreased significantly while aspartic acid and serine were increased in the BCGΔDosR. GraphPad Prism 5.0 was used to analyze the significance by a two-tailed Student’s *t*-test. The asterisk represents significant difference (*P < 0.05; **P < 0.01; ***P < 0.001).

**Figure 4:**
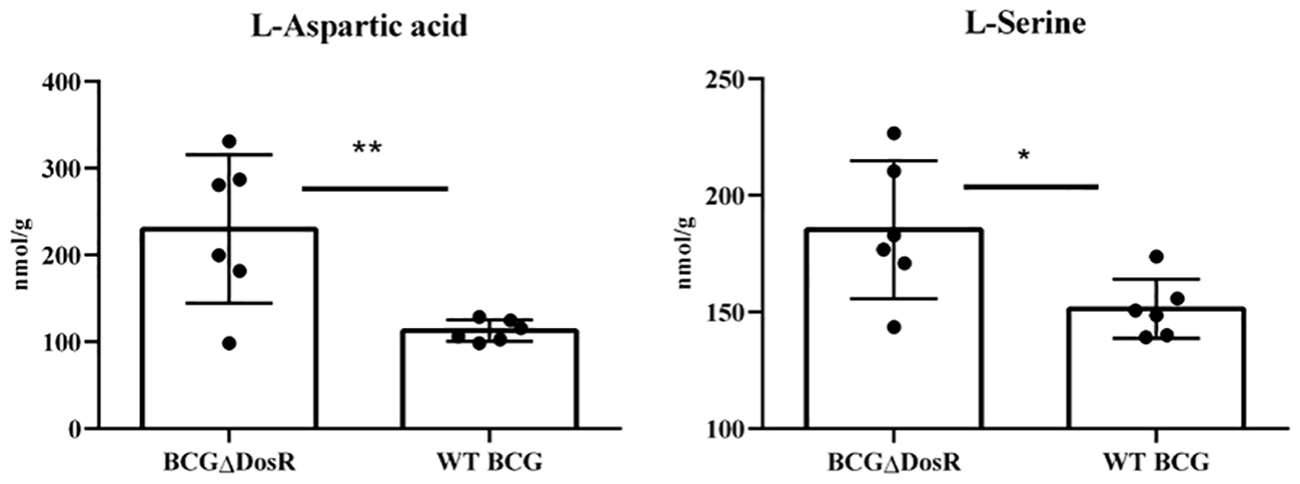
Amino acid metabonomic analysis. The concentration of amino acids in wild-type BCG (WT BCG) and DosR deletion strains (BCGΔDosR) was analyzed by targeted metabonomics, and the concentration of L-Aspartic acid and L-Serine increased significantly in BCGΔDosR. The results were analyzed with GraphPad Prism using a two-tailed Student’s *t*-test. Asterisks represent significant difference (*P < 0.05; **P < 0.01).

Amino acids, serving as crucial source of carbon and nitrogen, provide essential energy and metabolic intermediates for Mtb. To assess the effect of the *dosR* gene deletion on amino acid concentrations, we performed a targeted metabonomic analysis. The results are presented in **Figure 3**. Out of the 22 common amino acids, seven exhibited significant decreases in concentration, when compared to the WT strain. Of note, histidine and lysine concentrations exhibited the most substantial differences between the WT BCG and the BCGΔDosR strains. However, it appears that the synthesis of L-aspartic acid and L-serine has been enhanced in the BCGΔDosR strains, resulting in the accumulation of these amino acids, as depicted in **Figure 4**. These results indicate an impact of the *dosR* gene deletion on Mtb’s amino acids composition.

### The suppressing role of DosR on ROS and DNA damage in BCG

**Figure 5:**
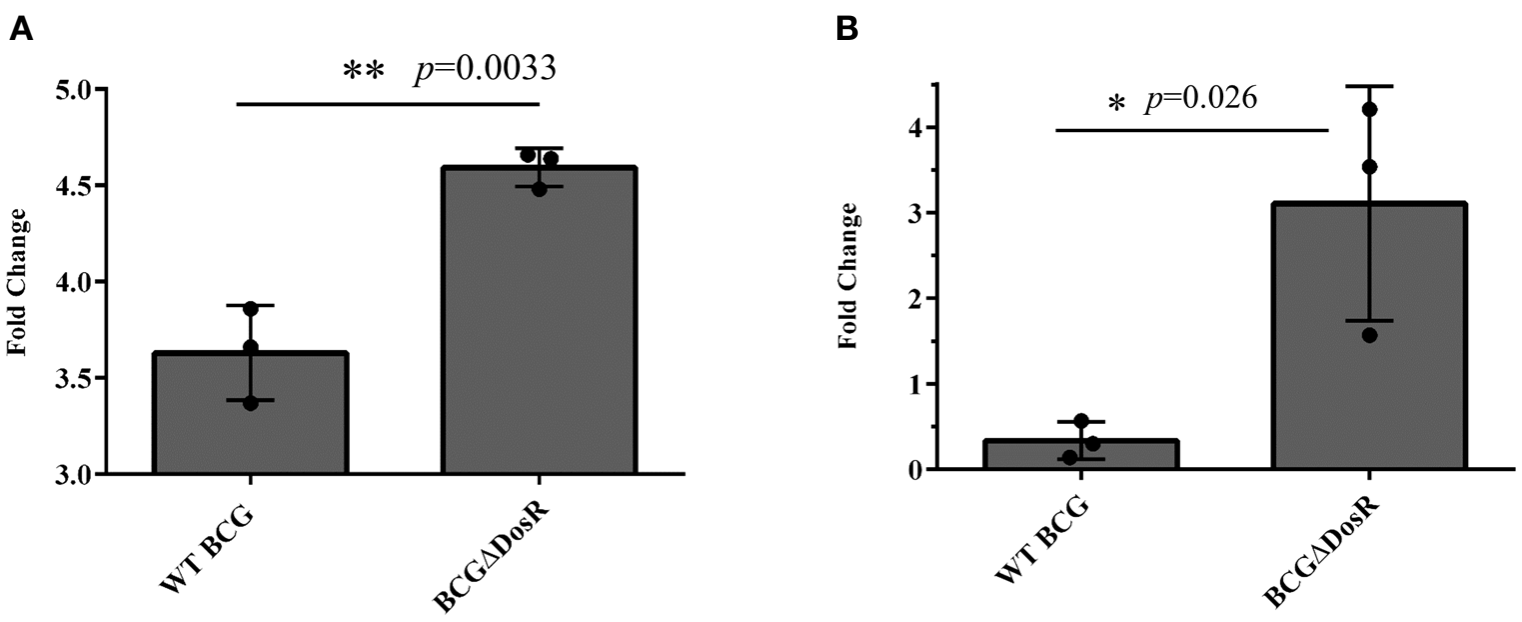
Assays for DosR regulation. **(A)** The evaluation of reactive oxygen species (ROS). ROS were measured in wild-type (WT) *Mycobacterium bovis* bacille Calmette-Guérin (BCG) and BCGΔDosR by flow cytometry; the bar chart shows the fold change relative to the untreated control. **(B)** The evaluation of DNA damage. The TUNEL assay measured DNA damage in WT BCG and BCGΔDosR. The bar chart shows the fold change relative to the untreated control. The analysis was conducted by GraphPad Prism using a two-tailed Student’s *t*-test. The asterisk represents a significant difference (*P < 0.05; **P < 0.01).

DosR, renowned for its role in ensuring survival, serves as the foundation for maintaining a stable redox state within the cell under hypoxia. The absence of DosR in BCG could lead to the production of ROS. To investigate this issue, we measured ROS and DNA damage in both WT BCG and BCG-lacking DosR (BCGΔDosR) using flow cytometry. The results shown in **Figure 5A** demonstrate an approximately 1.26-fold increase in ROS accumulation in the BCGΔDosR strain, compared to the WT BCG strain. Furthermore, **Figure 5B** presents a significant rise in DNA damage proportion when BCG lacked the *dosR* gene, exhibiting around a threefold increase in BCGΔDosR, compared to WT BCG. These results demonstrate that DosR inhibits ROS production and DNA damage, thereby strengthening its intracellular viability.

### Identification of potential novel *in-vivo* binding sites for DosR protein

**Figure 6:**
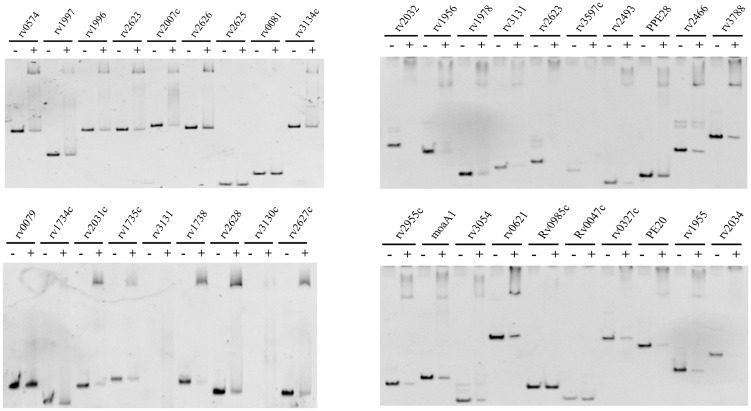
EMSA assay. EMSA identified the potential target genes of transcription analysis for DosR. The promoter DNA of the corresponding genes (3 nM) were reacted with 4 μM DosR and then subjected to gel analysis.

We selected 37 genes with the largest transcriptional differences, based on the results of transcriptome analysis. To assess their binding ability to the DosR protein using EMSA, we amplified the promoters of these selected genes. DosR demonstrated superior binding ability with 34 gene promoter regions within the DosR regulon, excluding *rv2625*, *rv0081*, and *rv0985c* (**Figure 6**). Additionally, DosR was confirmed to bind the promoter of *rv1955*, a gene previously considered unrelated to the DosR regulon, and thought to encode toxins. The promoters of *rv1978*, *rv0327c*, and *rv3054* also showed binding ability with DosR. In addition to the members of DosR regulon, potential targets and new binding sites may also be regulated by DosR.

## Discussion

In this study, we attempted to understand the relationship between BCG survival and transcriptome sequencing or proteomic change, by knocking out the *dosR* gene of *M. bovis* BCG. Transcription and expression of the *dosR* gene were downregulated in the mutant *ΔdosR*, based on the results of transcriptomic and proteomic analyses, DosR functions as a response regulator of the two-component system, DosR–DosS. This system induces the expression of the dormancy regulon in *M. bovi*s, playing a pivotal role in bacterial adaptation to hypoxia. Specifically, under hypoxic conditions, phosphorylated DosR interacts with multiple-binding sites within the promoter regions of target genes, regulating the expression of approximately 48 genes in H37Rv (Chauhan et al., 2011; Das et al., 2020). RNA-seq evaluation demonstrated that 104 genes were regulated by DosR. Ninety-nine of these genes were found to be downregulated in the mutant *ΔdosR*. Downregulation of 30 genes in this group was reported in previous studies (Chauhan et al., 2011; Das et al., 2020).

Chromosomal toxin/antitoxin (TA) systems are widespread genetic elements among bacteria (Rosendahl et al., 2020). In the genome of Mtb, there are approximately 88 putative TA systems (Ramage et al., 2009). These systems induce dormancy by inactivating essential metabolic functions, such as protein and ATP production (Jayaraman, 2008; Williams J. J. et al., 2011; Kwan et al., 2013). Different toxins and antitoxin systems were induced in response to specific stress conditions. For instance, as part of a type II toxin-antitoxin system, VapB38 (virulence associated protein) may be involved in streptomycin resistance and was also downregulated (−1.3 log_FC_) (Gupta et al., 2017; Sharrock et al., 2018). *HigB/HigA* (*higB*: *JTY_RS10150*, *higA*: *JTY_RS10155*) is crucial for preventing premature antitoxin degradation (Bordes et al., 2016). Both *higA* (−3.3 logFC) and *higB* (−3.9 logFC) were downregulated in the *ΔdosR* mutant. The *HigB/HigA* system features a reverse gene arrangement, with the toxin gene (*higB*) located upstream of the antitoxin gene (*higA*) (Tian et al., 1996), a pattern also observed in strain 172. Four genes of the *moaA1*-*moaD1* cluster (Molybdenum cofactor biosynthesis protein cluster) were found to be downregulated in the mutant strain. Molybdenum cofactor biosynthesis is associated with pathogenesis and hypoxic persistence (Mehra and Kaushal, 2009; Williams M. J. et al., 2011). Five *PE/PPE* genes, including *PE20*, *PE22*, *PPE22*, *PPE28*, and *PPE36*, were downregulated in the *ΔdosR* mutant. PE/PPE antigens play a fundamental role in host adaptation in many pathogenic species of mycobacteria, involving T-cell recognition and autophagy. Exposure of Mtb to various stress conditions affects the expression of *PE/PPE* genes, potentially aiding bacteria in adapting to the host during infection.

In the transcriptome, DosR negatively regulates the expression of five genes in BCG. Two of them, *JTY_RS20800* and *JTY_RS04510*, were annotated as hypothetical proteins, and gene *JTY_RS10315* was annotated as *cmtR*. Additionally, two other genes, *JTY_RS10490* and *JTY_RS04505*, were identified as membrane proteins and exhibited homology with *rv2025c* and *rv0849*. *Rv2025c* is downregulated in the H37Rv Δ*cnpB* strain, and gene *cnpB* controls expression of the CRISPR-Cas systems (Zhang et al., 2018). *Rv0849* belongs to the drug resistance type of antibiotic efflux pumps (Balganesh et al., 2012), while CmtR, a member of the ArsR/SmtB family, acts as a transcriptional sensor for metal toxicity (Campbell et al., 2007; Mishra et al., 2019). The large number of metal-sensing repressors from ArsR/SmtB family suggests that divalent and/or heavy metal adaptation may play an important role in the physiology and/or pathogenesis of the tubercle bacillus (He et al., 2011). *JTY_RS00450* (*Rv0081*) was downregulated in the *ΔdosR* mutant. Rv0081, a member of the ArsR/SmtB family of metal-dependent transcriptional repressors, is positively regulated by DosR through specific binding to recognition sequences (Campbell et al., 2007). The study’s results showed that DosR regulatory mechanism varies with different heavy metals.

Proteomic analysis demonstrated that expression of 179 genes was regulated by DosR (**Supplementary Table S3**). Thirty-seven proteins were identified as metabolite interconversion enzymes, while eight proteins were categorized as protein-modifying enzymes. Some virulence-associated proteins were downregulated in the mutant strain *ΔdosR*, including VapC4, VapC12, VapC37, VapC44, and VapC45. Surface-exposed unusual lipids containing phthiocerol and phenolphthiocerol are unique to the cell walls of slow-growing pathogenic mycobacteria. These lipids are believed to play important roles in host-pathogen interactions. The disruption of polyketide synthase (PKS) genes *ppsB* and *ppsC* in BCG leads to the cessation of the phthiocerol dimycocerosates and structurally related phenolic glycolipids production (Azad et al., 1997). *IniA* mediates TB drug-resistance through fission activity to maintain plasma membrane integrity, and *IniC* may regulate *IniA* activity and/or form hetero-oligomers with *IniA* (Wang et al., 2019). Both *IniA* and *IniC* were downregulated in the *ΔdosR* mutant. Phenolphthiocerol synthesis type-I PKS (PpsA, PpsB, and PpsC) was downregulated in the *ΔdosR* mutant.

Proteomic analysis revealed that three ribosome proteins, RpmA, RpmH, and RpmI, were downregulated in the *ΔdosR* mutant. Secreted PE/PPE proteins, which are associated with the mycobacterial outer membrane and have been believed to be involved in interacting with the host immune system (Delogu et al., 2017), were also affected. PE16, PPE21, and PPE69 were downregulated, while PPE32 was upregulated in the *ΔdosR* mutant. The secreted protein PtpB, known for its pivotal role in the pathogen`s interaction with the host cell (Koul et al., 2000), and its contribution to mycobacterial survival within its host (Rawls et al., 2010), was found to be upregulated in the *ΔdosR* mutant. In both transcriptomic and proteomic experiments, eight genes, including *dosR* and *dosS* (members of the two-component system), were downregulated in the *ΔdosR* mutant. The genes *hspX*/*acr* and *hrp*1 are known to be regulated by the *dosR* gene (Chauhan et al., 2011; Das et al., 2020). Additionally, three other genes, *JTY_RS00440*, *JTY_RS00445*, and *JTY_RS10505*, were homologous to three *H37Rv* genes (**Supplementary Table S2**) that are also regulated by *dosR* (Chauhan et al., 2011; Das et al., 2020). Furthermore, gene *JTY_RS03920* (*rv0744c*), encoding a transcriptional regulatory protein crucial for bacterial persistence, was found to be downregulated in the *ΔdosR* mutant (Talaat et al., 2004; Gautam et al., 2015).

In the targeted metabonomic analysis of amino acids, significant decreases were observed in the concentrations of specific amino acids, including 1-methy-L-histidine, glycine, L-histidine, lysine, and L-glutamine. Mtb meets its metabolic requirements within the host by either absorbing amino acids from the surrounding environment or synthesizing them intracellularly. In the context of amino acids, histidine serves as an illustrative example. Mtb initiates *de-novo* synthesis of histidine to bolster resistance mechanisms during infection. The *de-novo* synthesis of histidine plays a crucial role in protecting Mtb from host immune responses, particularly against IFN-γ–mediated histidine deficiency (Xu et al., 2007; Dwivedy et al., 2021). Biosynthetic genes for the specific form of lysine, diaminopimelic acid (DAP), are widespread among bacteria. Lysine is an essential amino acid for protein synthesis. For gram-positive bacteria, lysine is also an important component for the synthesis of peptidoglycan of the cell wall. Amino acids are also critical in the utilization of nitrogen by Mtb. Glutamine and serine have been identified as sources of nitrogen during macrophage infection (Lofthouse et al., 2013). The knockout of DosR leads to changes in amino acids, suggesting that DosR may directly or indirectly regulate amino acid synthesis and metabolism. In our integrated analysis of multiple omics data, we observed that DosR did not directly regulate the *de-novo* synthesis of amino acids under aerobic conditions. However, the transcriptional levels of several regulatory proteins associated with amino acid synthesis and genes responsive to nutrient deficiency conditions were found to be altered. Notably, we identified potential regulatory connections with TrcR, PhoR, and SenX3, indicating their potential involvement in the adaptation to hypoxia and reactivation processes.

Generally, DosR is essential for maintaining redox balance. Previous studies have indicated that the absence of DosR results in a significant decrease in both NAD and NADH levels (Leistikow et al., 2010). Simultaneously, being intermediate products of aerobic respiration, Mtb, and *Staphylococcus aureus* are sensitive to ROS. Our study revealed that the deletion of DosR caused a significant increase in the accumulation of ROS in Mtb. Additionally, NAD(P) was found to be essential for immune response, DNA repair, and multiple physiological processes, including redox reactions. Transcription analysis revealed a significant downregulation of NADH dehydrogenase and NAD(P)H nitroreductase, including *rv1812c*, *rv2032*, and *rv3131* genes. These three genes, part of the dormancy regulon (Bartek et al., 2009), include NADH dehydrogenase, crucial in the Mtb’s respiratory chain. Mutations in these genes can impair intracellular growth of TB bacteria (Vilcheze et al., 2018). In addition, the presence of NAD(P)H nitroreductase Rv3131 not only enhances the expression of proinflammatory factors like IL-6, TNF-α, and IL-12 but also represents a potential drug target (Dong et al., 2022). One can infer that an observed increase in intracellular ROS could be caused by the exchange of NAD/NADH. The regulatory role of DosR in Mtb may be more complex and extensive than previously thought.

## Data availability statement

The datasets presented in this study can be found in online repositories. The names of the repository/repositories and accession number(s) can be found in the article/**Supplementary Material**.

## Author contributions

YC: Methodology, Writing – original draft. GD: Funding acquisition, Resources, Writing – review & editing. HW: Investigation, Data curation, Software, Writing – review & editing. YT: Data curation, Formal analysis, Writing – review & editing. ML: Validation, Methodology, Writing – review & editing. SL: Conceptualization, Funding acquisition, Supervision, Writing – review & editing. NS: Conceptualization, Writing – review & editing.

## References

[B1] Azad A. K. Sirakova T. D. Fernandes N. D. Kolattukudy P. E. (1997). Gene knockout reveals A novel gene cluster for the synthesis of A class of cell wall lipids unique to pathogenic mycobacteria. J. Biol. Chem. 272, 16741–16745. doi: 10.1074/jbc.272.27.16741 9201977

[B2] Balganesh M. Dinesh N. Sharma S. Kuruppath S. Nair A. V. Sharma U. (2012). Efflux pumps of *Mycobacterium tuberculosis* play A significant role in antituberculosis activity of potential drug candidates. Antimicrob. Agents Chemother. 56, 2643–2651. doi: 10.1128/AAC.06003-11 22314527 PMC3346595

[B3] Bardarov S. Bardarov S. Pavelka M. S. Sambandamurthy V. Larsen M. Tufariello J.. (2002). Specialized transduction: an efficient method for generating marked and unmarked targeted gene disruptions in mycobacterium tuberculosis, *M. Bovis Bcg* and *M. Smegmatis* . Microbiology 148, 3007–3017. doi: 10.1099/00221287-148-10-3007 12368434

[B4] Bartek I. L. Rutherford R. Gruppo V. Morton R. A. Morris R. P. Klein M. R.. (2009). The Dosr regulon of *M. Tuberculosis* and antibacterial tolerance. Tuberc. (Edinb) 89, 310–316. doi: 10.1016/j.tube.2009.06.001 PMC2718728 19577518

[B5] Bordes P. Sala A. J. Ayala S. Texier P. Slama N. Cirinesi A. M.. (2016). Chaperone addiction of toxin-antitoxin systems. Nat. Commun. 7, 13339. doi: 10.1038/ncomms13339 27827369 PMC5105189

[B6] Campbell D. R. Chapman K. E. Waldron K. J. Tottey S. Kendall S. Cavallaro G.. (2007). Mycobacterial cells have dual nickel-cobalt sensors: sequence relationships and metal sites of metal-responsive repressors are not congruent. J. Biol. Chem. 282, 32298–32310. doi: 10.1074/jbc.M703451200 17726022 PMC3145109

[B7] Chakaya J. Khan M. Ntoumi F. Aklillu E. Fatima R. Mwaba P.. (2021). Global tuberculosis report 2020 - reflections on the global Tb burden, treatment and prevention efforts. Int. J. Infect. Dis. 113 Suppl 1, S7–S12. doi: 10.1016/j.ijid.2021.02.107 33716195 PMC8433257

[B8] Chauhan S. Sharma D. Singh A. Surolia A. Tyagi J. S. (2011). Comprehensive insights into *Mycobacterium tuberculosis* Devr (Dosr) regulon activation switch. Nucleic Acids Res. 39, 7400–7414. doi: 10.1093/nar/gkr375 21653552 PMC3177182

[B9] Crowther R. R. Qualls J. E. (2020). Metabolic regulation of immune responses to *Mycobacterium tuberculosis*: A spotlight on L-arginine and L-tryptophan metabolism. Front. Immunol. 11, 628432. doi: 10.3389/fimmu.2020.628432 33633745 PMC7900187

[B10] Cui Y. Dang G. Wang H. Tang Y. Lv M. Zang X.. (2022). Dosr regulates the transcription of the arginine biosynthesis gene cluster by binding to the regulatory sequences in mycobacterium bovis bacille calmette-guerin. DNA Cell Biol. 41 (12). doi: 10.1089/dna.2022.0282 36394437

[B11] Das J. Mapder T. Chattopadhyay S. Banik S. K. (2020). Computational study of parameter sensitivity in Devr regulated gene expression. PloS One 15, E0228967. doi: 10.1371/journal.pone.0228967 32053690 PMC7018068

[B12] Delogu G. Brennan M. J. Manganelli R. (2017). Pe and Ppe genes: A tale of conservation and diversity. Adv. Exp. Med. Biol. 1019, 191–207. doi: 10.1007/978-3-319-64371-7_10 29116636

[B13] Dong W. Z. Shi J. Chu P. Liu R. M. Wen S. A. Zhang T. T.. (2022). The putative Nad(P)H nitroreductase, Rv3131, is the probable activating enzyme for metronidazole in mycobacterium tuberculosis. BioMed. Environ. Sci. 35, 652–656. doi: 10.3967/bes2022.085 35945181

[B14] Dwivedy A. Ashraf A. Jha B. Kumar D. Agarwal N. Biswal B. K. (2021). *De novo* histidine biosynthesis protects mycobacterium tuberculosis from host Ifn-gamma mediated histidine starvation. Commun. Biol. 4, 410. doi: 10.1038/s42003-021-01926-4 33767335 PMC7994828

[B15] Gao X. Wu C. He W. Wang X. Li Y. Wang Y.. (2019). Dosr antigen Rv1737c induces activation of macrophages dependent on the Tlr2 pathway. Cell Immunol. 344, 103947. doi: 10.1016/j.cellimm.2019.103947 31326120

[B16] Gautam U. S. Mehra S. Kaushal D. (2015). *In-vivo* gene signatures of *Mycobacterium tuberculosis* in C3heb/Fej mice. PloS One 10, E0135208. doi: 10.1371/journal.pone.0135208 26270051 PMC4535907

[B17] Gupta A. Venkataraman B. Vasudevan M. Gopinath Bankar K. (2017). Co-expression network analysis of toxin-antitoxin loci in mycobacterium tuberculosis reveals key modulators of cellular stress. Sci. Rep. 7, 5868. doi: 10.1038/s41598-017-06003-7 28724903 PMC5517426

[B18] Harding E. (2020). Who global progress report on tuberculosis elimination. Lancet Respir. Med. 8, 19. doi: 10.1016/S2213-2600(19)30418-7 31706931

[B19] Hasenoehrl E. J. Rae Sajorda D. Berney-Meyer L. Johnson S. Tufariello J. M. Fuhrer T.. (2019). Derailing the aspartate pathway of *Mycobacterium tuberculosis* to eradicate persistent infection. Nat. Commun. 10, 4215. doi: 10.1038/s41467-019-12224-3 31527595 PMC6746716

[B20] He H. Bretl D. J. Penoske R. M. Anderson D. M. Zahrt T. C. (2011). Components of the Rv0081-Rv0088 locus, which encodes A predicted formate hydrogenlyase complex, are coregulated by Rv0081, Mpra, and Dosr in Mycobacterium tuberculosis. J. Bacteriol. 193, 5105–5118. doi: 10.1128/JB.05562-11 21821774 PMC3187382

[B21] Honaker R. W. Leistikow R. L. Bartek I. L. Voskuil M. I. (2009). Unique roles of Dost and Doss in Dosr regulon induction and *Mycobacterium tuberculosis* dormancy. Infect. Immun. 77, 3258–3263. doi: 10.1128/IAI.01449-08 19487478 PMC2715697

[B22] Jayaraman R. (2008). Bacterial persistence: some new insights into an old phenomenon. J. Biosci. 33, 795–805. doi: 10.1007/s12038-008-0099-3 19179767

[B23] Kim M. J. Park K. J. Ko I. J. Kim Y. M. Oh J. I. (2010). Different roles of doss and dost in the hypoxic adaptation of mycobacteria. J. Bacteriol. 192, 4868–4875. doi: 10.1128/JB.00550-10 20675480 PMC2944544

[B24] Koul A. Choidas A. Treder M. Tyagi A. K. Drlica K. Singh Y.. (2000). Cloning and characterization of secretory tyrosine phosphatases of *Mycobacterium tuberculosis* . J. Bacteriol. 182, 5425–5432. doi: 10.1128/JB.182.19.5425-5432.2000 10986245 PMC110985

[B25] Kwan B. W. Valenta J. A. Benedik M. J. Wood T. K. (2013). Arrested protein synthesis increases persister-like cell formation. Antimicrob. Agents Chemother. 57, 1468–1473. doi: 10.1128/AAC.02135-12 23295927 PMC3591907

[B26] Leistikow R. L. Morton R. A. Bartek I. L. Frimpong I. Wagner K. Voskuil M. I. (2010). The *Mycobacterium tuberculosis* Dosr regulon assists in metabolic homeostasis and enables rapid recovery from nonrespiring dormancy. J. Of Bacteriol. 192, 1662–1670. doi: 10.1128/JB.00926-09 20023019 PMC2832541

[B27] Lofthouse E. K. Wheeler P. R. Beste D. J. Khatri B. L. Wu H. Mendum T. A.. (2013). Systems-based approaches to probing metabolic variation within the *Mycobacterium tuberculosis* complex. PloS One 8, E75913. doi: 10.1371/journal.pone.0075913 24098743 PMC3783153

[B28] Mehra S. Kaushal D. (2009). Functional genomics reveals extended roles of the *Mycobacterium tuberculosis* stress response factor Sigmah. J. Bacteriol. 191, 3965–3980. doi: 10.1128/JB.00064-09 19376862 PMC2698404

[B29] Mishra R. Kohli S. Malhotra N. Bandyopadhyay P. Mehta M. Munshi M.. (2019). Targeting redox heterogeneity to counteract drug tolerance in replicating *Mycobacterium tuberculosis* . Sci. Transl. Med. 11 (518). doi: 10.1126/scitranslmed.aaw6635 PMC7212044 31723039

[B30] Park H. D. Guinn K. M. Harrell M. I. Liao R. Voskuil M. I. Tompa M.. (2003). Rv3133c/Dosr is A transcription factor that mediates the hypoxic response of *Mycobacterium tuberculosis* . Mol. Microbiol. 48, 833–843. doi: 10.1046/j.1365-2958.2003.03474.x 12694625 PMC1992516

[B31] Qualls J. E. Neale G. Smith A. M. Koo M. S. Defreitas A. A. Zhang H.. (2010). Arginine usage in mycobacteria-infected macrophages depends on autocrine-paracrine cytokine signaling. Sci. Signal 3, Ra62. doi: 10.1126/scisignal.2000955 20716764 PMC2928148

[B32] Ramage H. R. Connolly L. E. Cox J. S. (2009). Comprehensive functional analysis of *Mycobacterium tuberculosis* toxin-antitoxin systems: implications for pathogenesis, stress responses, and evolution. PloS Genet. 5, E1000767. doi: 10.1371/journal.pgen.1000767 20011113 PMC2781298

[B33] Rawls K. A. Grundner C. Ellman J. A. (2010). Design and synthesis of nonpeptidic, small molecule inhibitors for the *Mycobacterium tuberculosis* protein tyrosine phosphatase Ptpb. Org. Biomol. Chem. 8, 4066–4070. doi: 10.1039/c0ob00182a 20644889 PMC3009555

[B34] Roberts D. M. Liao R. P. Wisedchaisri G. Hol W. G. Sherman D. R. (2004). Two sensor kinases contribute to the hypoxic response of *Mycobacterium tuberculosis* . J. Biol. Chem. 279, 23082–23087. doi: 10.1074/jbc.M401230200 15033981 PMC1458500

[B35] Rosendahl S. Tamman H. Brauer A. Remm M. Horak R. (2020). Chromosomal toxin-antitoxin systems in pseudomonas putida are rather selfish than beneficial. Sci. Rep. 10, 9230. doi: 10.1038/s41598-020-65504-0 32513960 PMC7280312

[B36] Rustad T. R. Sherrid A. M. Minch K. J. Sherman D. R. (2009). Hypoxia: A window into *Mycobacterium tuberculosis* latency. Cell Microbiol. 11, 1151–1159. doi: 10.1111/j.1462-5822.2009.01325.x 19388905

[B37] Saleh S. Staes A. Deborggraeve S. Gevaert K. (2019). Targeted proteomics for studying pathogenic bacteria. Proteomics 19, E1800435. doi: 10.1002/pmic.201800435 31241236

[B38] Sharrock A. Ruthe A. Andrews E. S. V. Arcus V. A. Hicks J. L. (2018). Vapc proteins from *Mycobacterium tuberculosis* share Ribonuclease sequence specificity but differ in regulation and toxicity. PloS One 13, E0203412. doi: 10.1371/journal.pone.0203412 30169502 PMC6118392

[B39] Shiloh M. U. Manzanillo P. Cox J. S. (2008). *Mycobacterium tuberculosis* senses host-derived carbon monoxide during macrophage infection. Cell Host Microbe 3, 323–330. doi: 10.1016/j.chom.2008.03.007 18474359 PMC2873178

[B40] Song N. Zhu Y. Cui Y. Lv M. Tang Y. Cui Z.. (2020). Vitamin B and vitamin C affect Dna methylation and amino acid metabolism in mycobacterium Bovis Bcg. Front. Microbiol. 11, 812. doi: 10.3389/fmicb.2020.00812 32390998 PMC7188828

[B41] Talaat A. M. Lyons R. Howard S. T. Johnston S. A. (2004). The temporal expression profile of *Mycobacterium tuberculosis* infection in mice. Proc. Natl. Acad. Sci. U.S.A. 101, 4602–4607. doi: 10.1073/pnas.0306023101 15070764 PMC384793

[B42] Tian Q. B. Hayashi T. Murata T. Terawaki Y. (1996). Gene product identification and promoter analysis of Hig locus of plasmid Rts1. Biochem. Biophys. Res. Commun. 225, 679–684. doi: 10.1006/bbrc.1996.1229 8753818

[B43] Tiwari S. Van Tonder A. J. Vilcheze C. Mendes V. Thomas S. E. Malek A.. (2018). Arginine-deprivation-induced oxidative damage sterilizes *Mycobacterium tuberculosis* . Proc. Natl. Acad. Sci. U.S.A. 115, 9779–9784. doi: 10.1073/pnas.1808874115 30143580 PMC6166831

[B44] Vesosky B. Rottinghaus E. K. Stromberg P. Turner J. Beamer G. (2010). Ccl5 participates in early protection against *Mycobacterium tuberculosis* . J. Leukoc. Biol. 87, 1153–1165. doi: 10.1189/jlb.1109742 20371596 PMC2872537

[B45] Vilcheze C. Weinrick B. Leung L. W. Jacobs W. R. Jr. (2018). Plasticity of *Mycobacterium tuberculosis* Nadh dehydrogenases and their role in virulence. Proc. Natl. Acad. Sci. U.S.A. 115, 1599–1604. doi: 10.1073/pnas.1721545115 29382761 PMC5816213

[B46] Voskuil M. I. Visconti K. C. Schoolnik G. K. (2004). *Mycobacterium tuberculosis* gene expression during adaptation to stationary phase and low-oxygen dormancy. Tuberculosis 84, 218–227. doi: 10.1016/j.tube.2004.02.003 15207491

[B47] Wang M. Guo X. Yang X. Zhang B. Ren J. Liu A.. (2019). Mycobacterial dynamin-like protein Inia mediates membrane fission. Nat. Commun. 10, 3906. doi: 10.1038/s41467-019-11860-z 31467269 PMC6715688

[B48] Williams J. J. Halvorsen E. M. Dwyer E. M. Difazio R. M. Hergenrother P. J. (2011). Toxin-antitoxin (Ta) systems are prevalent and transcribed in clinical isolates of *Pseudomonas aeruginosa* and methicillin-resistant Staphylococcus aureus. FEMS Microbiol. Lett. 322, 41–50. doi: 10.1111/j.1574-6968.2011.02330.x 21658105 PMC3184004

[B49] Williams M. J. Kana B. D. Mizrahi V. (2011). Functional analysis of molybdopterin biosynthesis in mycobacteria identifies A fused molybdopterin synthase in mycobacterium tuberculosis. J. Bacteriol. 193, 98–106. doi: 10.1128/JB.00774-10 20971904 PMC3019926

[B50] Xu Y. Labedan B. Glansdorff N. (2007). Surprising arginine biosynthesis: A reappraisal of the enzymology and evolution of the pathway in microorganisms. Microbiol. Mol. Biol. Rev. 71, 36–47. doi: 10.1128/MMBR.00032-06 17347518 PMC1847373

[B51] Zhang Y. Yang J. Bai G. (2018). Regulation of the Crispr-associated genes by Rv2837c (Cnpb) via an Orn-like activity in tuberculosis complex mycobacteria. J. Bacteriol. 200 (8), e00743-17. doi: 10.1128/JB.00743-17 29378893 PMC5869477

