# Supplementary material for: DosR’s multifaceted role on Mycobacterium bovis BCG revealed through multi-omics
Source: Front Cell Infect Microbiol. 2023 Nov 21;13:1292864. doi: 10.3389/fcimb.2023.1292864 (PMC10703047; doi:10.3389/fcimb.2023.1292864)
Supplement: Supplementary file 1 [file DataSheet_1.docx]

**DosR's Multifaceted Role on *Mycobacterium bovis* BCG Revealed Through Multi-Omics**

Yingying Cui^1^, Guanghui Dang^1^, Hui Wang^1^, Yiyi Tang^1^, Mingyue Lv^1^, Siguo Liu^1^*, Ningning Song^1,2,3^*

^1^ State Key Laboratory for Animal Disease Control and Prevention, Division of Bacterial Diseases, Harbin Veterinary Research Institute, Chinese Academy of Agricultural Sciences, NO.678, Haping Street, Harbin 150069, PR China

^2^School of life science and technology, Weifang Medical University, NO. 7166, Bao Tong West Street, Weifang 261053, China

^3^ Weifang Key Laboratory of Respiratory Tract Pathogens and Drug Therapy，Weifang ,China

*Corresponding author: Siguo Liu, Ningning Song

E-mail:  liusiguo@caas.cn

songningning@wfmc.edu.cn

**Table S1. Primers used in this study**

| **Primers** | **Sequence (5`-3`)** | **Function** |
| --- | --- | --- |
| DosR-F | CGCGGATCCGGTAAAGGTCTTCTTGGTCGA (BamH I) | Amplification for *dosR* |
| DosR-R | GGGAATTCCATATGTGGTCCATCACCGGGTG (Nde I) | Amplification for *dosR* |
| 3133RFP-F | TTTTTTTTCCAT**AGA**TTGGTCGACCAAGAAGACCTTTACC | Amplification for right arm |
| 3133RFP-R | TTTTTTTTCCAT**CTT**TTGGTGTACGTCATCGATCCGTCCC | Amplification for right arm |
| 3133LFP-F | TTTTTTTTCCAT**AAA**TTGGGGACCAGAACCTCGTCGTCGT | Amplification for left arm |
| 3133LFP-R | TTTTTTTTCCAT**TTC**TTGGGCCACCCGGTGATGGACCATG | Amplification for left arm |
| Hyg-F | GGCCCGTACCCTGTGAATAGAGGTCCGCT | Identification of *Hyg* |
| Hyg-R | CGTTGAGATGCAGTTGCACCAGGCTGTAGCG | Identification of *Hyg* |
| sacB-F | CGGCACTGTCGCAAACTATC | Identification of *sacB* |
| sacB-R | GCTTTTGCCATAGTATGCTTTGT | Identification of *sacB* |
| 3133-out-F | TGACGCGCCTTAGCCTGCTGG | Identification of BCGΔDosR |
| 3133-out-R | AGCTCGGGCAGTGGTCGTTGG | Identification of BCGΔDosR |
| 3133-in-F | TCCGCTGGCACCGGCGAGAAT | Identification of BCGΔDosR |
| 3133-in-R | TTCGGTCGCCGAGGCGATGGCCA | Identification of BCGΔDosR |
| rv0574c-F | TCCGGACCACCGCCGCCTTCA | EMSA |
| rv0574c-R | AAGCAAGCCAGTGCGGCGAAG | EMSA |
| rv1997-F | CAGTGCCGGCCTGAGGGCCT | EMSA |
| rv1997-R | TCACCTCACCTCGCAGCCAG | EMSA |
| rv1996-F | CGAGACACCCTGGCCCAAGAC | EMSA |
| rv1996-R | TGGCGTCCCCTCTGCATGGGT | EMSA |
| rv2623-F | TCGAACACGTTGTCACCGCTT | EMSA |
| rv2623-R | CGCGGTCCTCCTGTCGTTGG | EMSA |
| rv2007c-F | AAACGGCAAGCTACCGGCCC | EMSA |
| rv2007c-R | CGGTCGATGCGCTCGGCTCTT | EMSA |
| rv2626-F | CGGGATCCTCCTCGAGTCGG | EMSA |
| rv2626-R | CCCGTCGCGAGGCTCATGTG | EMSA |
| rv2625-F | AGCGGGATCGCATCACGCAT | EMSA |
| rv2625-R | TGCTCGCCCATGGCCCTCGC | EMSA |
| rv0081-F | GCTGACTCGCGTACGCCGTA | EMSA |
| rv0081-R | AGCGGTTCGGACTCCACCGC | EMSA |
| rv3134c-F | CGCCGTCTCCTTCGCTGGGG | EMSA |
| rv3134c-R | GACGCCGTGCCGGCCGCATC | EMSA |
| rv0079-F | GCGTCATCCTCCACGCGTCGATA | EMSA |
| rv0079-R | CTGCTCGGCTTTCGATTGTGG | EMSA |
| rv1734c-F | GGTGATCGTGAGGTCCGATA | EMSA |
| rv1734c-R | CCAGCCGATGTAGCTGAGATAC | EMSA |
| rv2031c-F | TTGATGCCTCCTAATCGATGG | EMSA |
| rv2031c-R | TCGCGTAAAGCACCCGATCC | EMSA |
| rv1735c-F | CGCCACCCAATAGGGCGG | EMSA |
| rv1735c-R | TCGATTTCGATGCGAGACACG | EMSA |
| rv3131-F | TCGTTAGGTGATTCATGGTC | EMSA |
| rv3131-R | GGAAATGGGTGTTCATGGCT | EMSA |
| rv1738-F | ACGCCCAGAAGTTGACCACC | EMSA |
| rv1738-R | GATGTTGCTCCCCTTTCCAG | EMSA |
| rv2628-F | GCAGAACTTGCCATGGCAGC | EMSA |
| rv2628-R | GCCGAAGTCCGTGTCCACTG | EMSA |
| rv3130c-F | GGTCAGCGCCTTCCCCGGTG | EMSA |
| rv3130c-R | GGAAATGGGTGTTCATGGCT | EMSA |
| rv2627c-F | GGCAGCAAAGCTAACCGCGT | EMSA |
| rv2627c-R | CGTTGCGTGGACATGCCG | EMSA |
| rv2955c-F | CTTGGAACTGCACCAGGTCC | EMSA |
| rv2955c-R | AACGAGCGAGTTTCAACGAT | EMSA |
| moaA1-F | GATTCCGCCCTAACAACGCG | EMSA |
| moaA1-R | GTAGGCGTACTCATTTTCCT | EMSA |
| rv3054-F | TGGTATCTGACACTGCTACG | EMSA |
| rv3054-R | GCTCGGCGCCGCTCACCTTT | EMSA |
| rv0621-F | CGGCTGCACCTCGAGGCAAA | EMSA |
| rv0621-R | AGGATGGCGAGCTCCAGCAT | EMSA |
| rv0047c-F | ACGTCAGCTACCCGTCTTGA | EMSA |
| rv0047c-R | TCGTCAACGTGCTTGCC | EMSA |
| rv0327c-F | GTTTGTCGCGGTTTGTG | EMSA |
| rv0327c-R | CTTTGAGCATGCGATGTCCT | EMSA |
| rv0985c-F | AGGTCCTTTGTTGGATATTT | EMSA |
| rv0985c-R | AGGTCCTTTGTTGGATATTT | EMSA |
| PE20-F | GATCCGACGAAGAACAAG | EMSA |
| PE20-R | TGGATCAGGGGGCGGCAC | EMSA |
| rv1955-F | TCGGTTCCATAGCGATC | EMSA |
| rv1955-R | TAAGTGGACACGAACCG | EMSA |
| rv2034-F | GGCGCTGAACGGGAAGG | EMSA |
| rv2034-R | GTGTCCGGCATGATCAA | EMSA |
| rv2032-F | CCCAAGACGAAGATCGA | EMSA |
| rv2032-R | GCCTTTGAAGCTGGGATC | EMSA |
| rv1956-F | CAGCCCTCGAAGTGACGGCC | EMSA |
| rv1956-R | TCGCCTCACCCATGGCAGCT | EMSA |
| rv1978-F | TCGTTAGGTGATTCATGGTC | EMSA |
| rv1978-R | GGAAATGGGTGTTCATGGCT | EMSA |
| rv3131-F | TCGAACACGTTGTCACCGCTT | EMSA |
| rv3131-R | CGCGGTCCTCCTGTCGTTGG | EMSA |
| rv2623-F | TCTTCGCCATTGGCTTACCC | EMSA |
| rv2623-R | TCGACCACACTCCAACTGAA | EMSA |
| rv3597c-F | ACAATTTGACGCGGTGCGGA | EMSA |
| rv3597c-R | CACATCGTCGTCAAGGTCAA | EMSA |
| rv2493-F | GCGGCGTCTGACCGCCCCGT | EMSA |
| rv2493-R | ATTCGGCAGCATCGCGACGG | EMSA |
| PPE28-F | CGAGCATGGCGCGGTCCTCT | EMSA |
| PPE28-R | TCGGTCACATCGAGAATGAT | EMSA |
| rv2466c-F | GCCACGGATCCATCTTCGGC | EMSA |
| rv2466c-R | TTTCTCGCTCACGTCCGCCG | EMSA |
| rv3788-F | GCCACGGATCCATCTTCGGC | EMSA |
| rv3788-R | TTTCTCGCTCACGTCCGCCG | EMSA |
| Rv0079-F | GAGGACGCTGTGGTCTATCG | qPCR |
| Rv0079-R | GAATGGCAGTCCATGTTCGC | qPCR |
| Rv0080-F | GCGATCCGTCCAGTCAATCA | qPCR |
| Rv0080-R | GGATCAAGGTCGTCGGCTTC | qPCR |
| Rv0744c-F | GCTGTTGACCGAACCGGATA | qPCR |
| Rv0744c-R | GTCAATACGTGCATGACCGC | qPCR |
| Rv2028c-F | GGCGAGCTGCTGGATTATCT | qPCR |
| Rv2028c-R | GATACTGCTGACCGACGACC | qPCR |
| DevR-F | GCTATCAGGCCTTACCGACC | qPCR |
| DevR-R | AACCGCGACACGTAGTTCTT | qPCR |
| DevS-F | AGGTCTTCGTCAACGGCATT | qPCR |
| DevS-R | CTCGAGTTGTTCGTCGGTGA | qPCR |
| Hrp1-F | CTAGACCCGAATACCGCCAC | qPCR |
| Hrp1-R | GCGGACCTGATGTTCTTCCA | qPCR |
| hspX-F | TTATGGTCCGCGATGGTCAG | qPCR |
| hspX-R | AATGCCCTTGTCGTAGGTGG | qPCR |

**
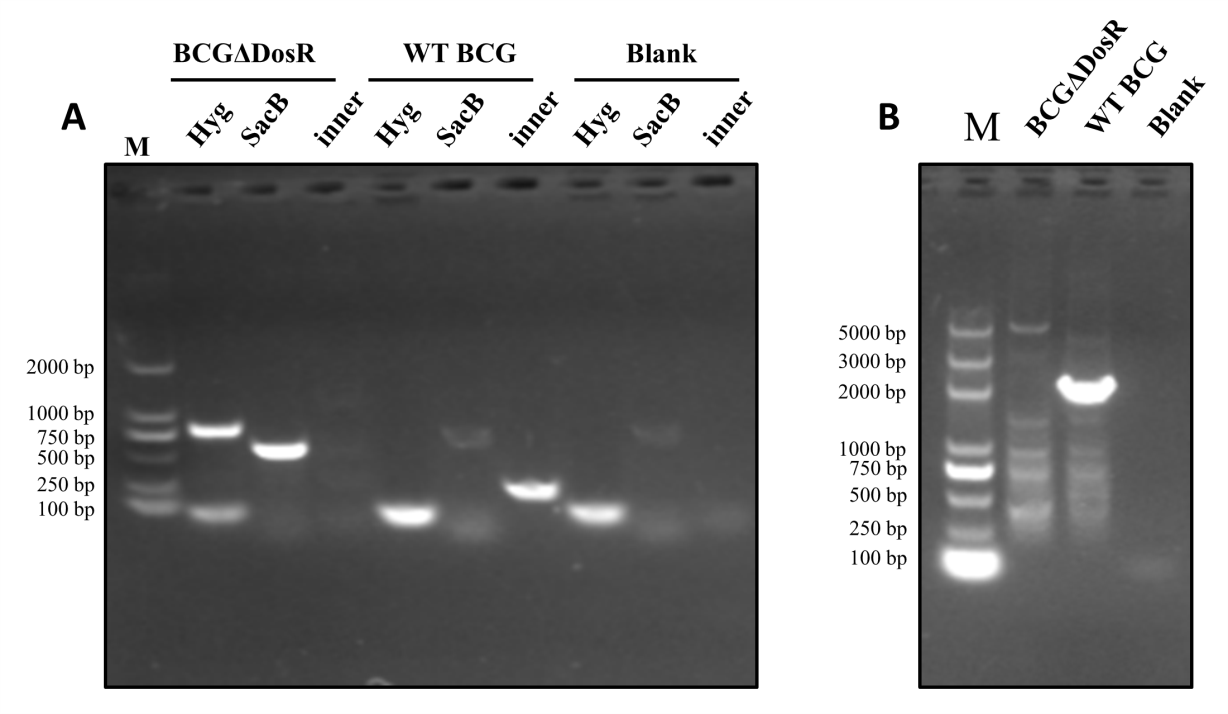
**

**Figure S1 Identification of BCGΔdosR by PCR**

(A) PCR identification of BCGΔDosR strain and wild BCG strain M: DL2000 DNA marker;1: Identification of *Hyg* gene of BCGΔDosR strain; 2: Identification of *sacB* gene of BCGΔDosR strain;3: Identification of internal gene of BCGΔDosR; 4. Identification of *Hyg* gene of wild strain; 5. Identification of *sacB* gene of wild strain; 6: Identification of internal gene of *dosR* of wild strain; 7-9: blank control.

(B) PCR identification of the external gene of BCGΔDosR strain and wild BCG strain M: DL5000 DNA marker; 1: Identification of external gene of BCGΔDosR; 2: Identification of external gene of wild strain.

**
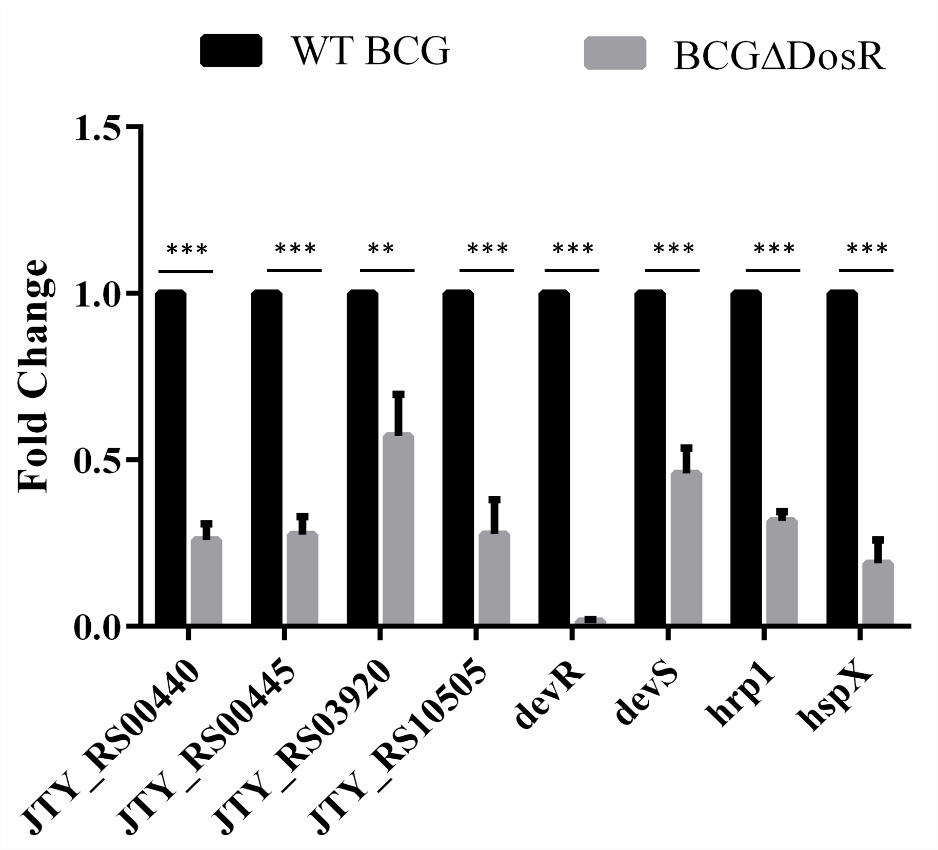
**

**Figure S2 q-PCR analysis of the genes in Table 1.**

The expression of the genes between WT BCG and BCGΔDosR strains were evaluated by quantitative PCR (qPCR). GraphPad Prism 5.0 was used for analysed the significance by a two-tailed Student’s *t*-test. The asterisk represents significant difference (*, *P* < 0.05; **, *P* < 0.01; ***, *P* < 0.001).
